# Use of the Analysis of the Volatile Faecal Metabolome in Screening for Colorectal Cancer

**DOI:** 10.1371/journal.pone.0130301

**Published:** 2015-06-18

**Authors:** Claire A Batty, Michael Cauchi, Célia Lourenço, John O Hunter, Claire Turner

**Affiliations:** 1 Dept. Life, Health & Chemical Sciences, The Open University, Walton Hall, Milton Keynes, United Kingdom; 2 Centre for Biomedical Engineering, Cranfield University, Cranfield, Bedfordshire, United Kingdom; 3 Gastroenterology Research Dept., Addenbrooke’s Hospital, Cambridge, United Kingdom; INSERM, FRANCE

## Abstract

Diagnosis of colorectal cancer is an invasive and expensive colonoscopy, which is usually carried out after a positive screening test. Unfortunately, existing screening tests lack specificity and sensitivity, hence many unnecessary colonoscopies are performed. Here we report on a potential new screening test for colorectal cancer based on the analysis of volatile organic compounds (VOCs) in the headspace of faecal samples. Faecal samples were obtained from subjects who had a positive faecal occult blood sample (FOBT). Subjects subsequently had colonoscopies performed to classify them into low risk (non-cancer) and high risk (colorectal cancer) groups. Volatile organic compounds were analysed by selected ion flow tube mass spectrometry (SIFT-MS) and then data were analysed using both univariate and multivariate statistical methods. Ions most likely from hydrogen sulphide, dimethyl sulphide and dimethyl disulphide are statistically significantly higher in samples from high risk rather than low risk subjects. Results using multivariate methods show that the test gives a correct classification of 75% with 78% specificity and 72% sensitivity on FOBT positive samples, offering a potentially effective alternative to FOBT.

## Introduction

Colorectal cancer is the fourth most common cancer in men and the 3rd most common cancer in women. There is significant international variation in the distribution of this cancer [1] and it is often regarded as a disease of Western industrialised countries. This strongly suggests that environmental factors may play a major role in the aetiology [2], and the highest incidence rates of colorectal cancer are located in North America, Europe and Oceania [1,3].

Despite the higher incidence of this cancer, mortality has been found to have decreased in 13 out of 29 countries [1]. The decrease is generally considered to be due to improvements in treatment, screening and earlier detection and was seen in longstanding economically stable countries such as the USA, Australia, France and the UK. Even better screening and detection may improve this further.

### Screening Programmes and Strategies

Many countries now choose to screen for this disease as a matter of course to obtain earlier diagnosis. There is growing evidence that screening asymptomatic people who are at an average risk of colorectal cancer means cancers are generally detected earlier, are more ‘curable’ and result in an overall reduction in mortality [4]. It is thought that 70–90% of cases of colorectal cancer arise from premalignant (adenomatous) polyps. They often have a stalk which consists of healthy tissue and allows them to be removed simply and completely by endoscopic snaring which enhances the need for adequate screening [5].

In the UK, much effort has been placed on setting up an adequate screening programme. On the advice of the National Screening Committee, the UK Department of Health carried out a demonstration pilot to test the feasibility of a national screening programme for colorectal cancer [6]; these were then expanded to further pilots and proved successful in their aim of bringing colorectal cancer screening into the UK’s National Health Service (NHS) [7]. It is now a nationally rolled out protocol where the NHS offer free screening to all men and women aged 60–69 in the UK every two years, and on request can be sent to anyone over 70 years of age [8].

In both the USA and the UK, the means of screening subjects are generally similar with initial screening being stool/faecal based tests, and then secondary structural based tests. In both the US and UK, the chosen faecal based tests is the faecal occult blood test (FOBT) and then the more invasive structural test is the colonoscopy/sigmoidoscopy [6, 7, 9, 10, 11].

The FOBT is designed to detect occult (i.e. not accompanied by discernible symptoms or signs) blood in the stool and works on the basis of the fact that large polyps and the actual colorectal cancers in the colon and rectum tend to bleed. The test requires the sampler to smear a card with faeces twice after a bowel movement, then repeat this for a further two bowel movements, making a total of six windows covered on the card. This is then sent in a hygienically sealed envelope back to the relevant clinic and is tested. A positive result is deemed to be when any of the windows return as ‘positive’ [12,13].

The benefits of this test include its relatively low invasiveness, low initial cost and the fact it requires few specialised resources. It is also possible to do this test at home easily, making it perfect for a large scale screening programme. It has also been proven to help reduce the incidence of colorectal cancer [4, 11, 14, 15]. However, the test is not specific for human haemoglobin and also does not take into account blood that may be from other sources, i.e. haemorrhoids [4] and peptic ulcers. Another problem is that the sensitivity and specificity of the test is variable. Its sensitivity has been reported at around 40% for cancer and only 24% for the detection of advanced adenoma or *in situ* carcinoma [16]. If the FOBT is used as a screening procedure for the general population, the predictive value of a positive test is no more than 5% to 10%. This means most patients who test positive will require an expensive, uncomfortable, confirmatory diagnostic procedure such as colonoscopy when in fact they do not have colorectal cancer [17]. Although FOBT is the most commonly used screening test, it does not offer the optimum requisites of a screening test, due to its lack of sensitivity and specificity. Another screening test is for abnormal DNA in a faecal sample. This abnormal DNA arises from mutations occurring in cancer or adenoma cells, which then may be shed into the faeces. Imperiale et al [18] compared this method with the FOBT and found that in a limited study, it proved to be more sensitive than FOBT with similar specificity.

### Use of biomarkers in screening

The human gut has a microbiome with a large bacterial population which can be commensal, but has also been shown to cause some detrimental effects. Mounting evidence has shown a relationship between infective agents and some human cancers. Certain mucosal associated bacterial species play an important role in the pathogenesis of colorectal cancer [19]. Metabolite profiling of volatile organic compounds (VOCs) of human colon cell lines including normal, and human cell carcinoma, offer not only biochemical phenotyping of normal and neoplastic colon tissue, but also differences in the volatile metabolome in different disease stages in comparison to the volatile metabolome of non-malignant colon epithelial cell lines [20,21].

The ‘scent’ of disease is becoming more prominent; and canine scent detection, i.e. use of dogs, has shown that the dogs are able to differentiate between samples from cancer patients and those without cancer. This suggests and reinforces that there are different ‘volatile compounds’ between cancer and non-cancer [22,23].

It was also reported that the pattern of breath VOCs differed between healthy controls and those diagnosed with colorectal cancer. Analysis showed an initial specificity of 83% with a sensitivity of 86% and an overall accuracy of 85%. When this then went through further validation testing on 25 subjects, 19 were correctly identified leading to 76% accuracy. This is encouraging for a more accurate screening test [24].

The characteristic patterns of VOCs in faeces have been reported for the many different causes of diarrhoea. As a potentially fast and convenient method, it opens up a new area for use as a non-invasive diagnostic tool. It can be performed repeatedly, can be applied to children including neonates as samples are easy to collect, and to patients with severe disease where more invasive procedures are not easily possible [25,26].

In this research, the difference in volatile profiles of high and low risk colorectal samples is investigated to see whether VOC analysis has potential for screening or diagnosis of colorectal cancer.

## Materials and Methods

### Sample acquisition

The cancer samples in this study were collected through the National NHS Bowel Cancer Screening Scheme. This service is offered nationally every 2 years to all males and females between the age of 60 and 69. Subjects received an invitation letter explaining the programme along with an information leaflet from the programme hub centre. Approximately 1 week later, the FOBT is sent out to the subject with step-by-step instructions for completing the test at home and then how to send the sample back to the hub lab. In the test, participants are sent out a cardboard envelope containing three windows on which may be smeared a thin sample of faeces. In the kit there is a spatula for this purpose together with clear instructions. The test is called Haemoccult by Beckman Coulter. A sample is taken on three separate days and once complete, the participant then seals the cardboard envelope and sends it back to the laboratory. The test card is then processed at the hub lab and the results were returned to the subject within 2 weeks.

Subjects who tested positive with FOBT screening and then accepted a colonoscopy were invited to participate in this research. These patients are requested to produce a further stool sample to bring with them in a wide plastic sterile container provided by the hospital. It was these samples which we studied.

After the colonoscopy, the patients were stratified into risk groups based on their histopathology. In total there were 7 classes as illustrated in Table 1.

**Table 1:** Risk Class Applied to Histological Results.

Class	Detail	Number of samples
1	Normal, nothing of concern found = **low risk**	31
2	Hyperplastic polyp	0
3	Adenoma—Polyp less than 10mm diameter	0
4	Adenoma—polyp more than 10mm diameter	0
5	High Grade Adenoma = **high risk**	31
6	Adenocarcinoma = **high risk**	
99	Other (for example IBD)	0

In this study, 31 samples were obtained from the ‘normal’ or low risk group (class 1) and 31 samples were obtained from the high risk groups (class 5 and 6). However, all samples were from individuals who had screened positive using the FOB test. After samples were obtained from patients, they were stored at -80°C until processing by selected ion flow tube mass spectrometry (SIFT-MS) as described below. The resulting data were then processed using multivariate statistical techniques.

### Ethics statement

Samples were taken under the National NHS bowel cancer screening scheme. Favourable ethical opinion was obtained for subsequent inclusion of suitable subjects in this research by Cambridgeshire 2 REC, REF 08/H0308/13, and written informed consent obtained from each subject and consent form then kept securely. All samples were anonymised prior to analysis and only the cancer status was known to the authors.

### Sample Preparation

Human faecal samples were removed from the -80°C freezer and 5g sub samples were weighed out. Each sub sample was placed into a Nalophan bag and sealed with a Swagelokconnector and tube. The bag was filled with hydrocarbon free air and placed in the incubator for 45 minutes.

### SIFT-MS analysis

Full details of how SIFT-MS may be used to analyse trace gases and volatile organic compounds may be found elsewhere [27], however, a brief explanation is warranted here. In SIFT-MS, precursor ions (H_3_O^+^, NO^+^ and O_2_^+^) are generated in a microwave discharge and the chosen ion is selected by a quadrupole mass filter. The selected ion is then injected into a fast flowing helium carrier gas, and down a flow tube. A sample is then introduced into the flow tube, and the precursor ion reacts with the trace gases and volatile organic compounds in the sample. The precursor and product ions in the carrier gas are separated in a second quadrupole mass spectrometer and subsequently counted in a detector. Data may be obtained through scanning a spectrum at a user-defined range of mass-to-charge ratio (m/z) values and quantification is carried out using a kinetics database stored in the instrument.

After incubation, sample bags in the incubator were connected to the SIFT-MS via the heated sampling line, and then analysed using each of the three precursor ions available in SIFT-MS and in the same order of H_3_O^+^ then NO^+^ and finally O_2_^+^. The m/z range was set from 10 to 140, and the total time for analysis using each precursor was 30 seconds.

Blank samples of background air were also analysed. Data were then transferred to the computer to be analysed for the range of compounds present in each sample.

### Statistical analysis

Data (in file S1 Table) were analysed using appropriate univariate and multivariate statistical techniques. Multivariate data analysis was performed by custom-built scripts written in MATLAB R2011a (MathWorks Inc., Nattick, USA) using functions from the PLS Toolbox (version 3.5, EigenVector Research Inc., USA).

The univariate technique involved the non-parametric Mann-Whitney U test analysis which was used because the data were not normally distributed. The analysis was performed using Excel and was chosen as this test does not assume normal distribution or that the variances of two populations are equal. It is also a useful technique for smaller sample groups and is based on the comparison of each observation from the first group with each observation from the second group. The data from each group are then individually compared together. It is also a useful technique for semi-quantitative data and has good probability of producing statistically significant results [28].

Prior to multivariate data analysis, data generated by the H_3_O^+^, NO^+^ and O_2_^+^ precursor ions were combined into one large dataset. The intensity values at the range of m/z values within the data were normalised against the intensity values of the H_3_O^+^ precursor ions (m/z value of 19). The m/z values pertaining to the known adducts (isotopologues) of the precursor ions were removed; these had the following m/z values: 19, 21, 30, 32, 34, 37, 39, 48, 55, 57, 66, 73, 75, and 91. Finally, m/z values pertaining to data columns consisting only of zeros were also removed.

Exploratory data analysis using principal components analysis [29] revealed no outlying samples.

Following feature selection via either the parametric student *t* test [30] or the non-parametric Wilcoxon T test [31], multivariate classification via partial least squares discriminant analysis (PLS-DA) was performed [32,33]. This is a supervised pattern recognition technique in which the computer is trained to recognise patterns in the data that will help distinguish between low and high risk patients. To ensure a robust and confident result was attained, a two-step bootstrapping process was employed in which the first step deduces an optimal model, and the second step evaluates the model. This was repeated 150 times to attain an average performance of all models created.,. The statistical significance of the classification accuracy was determined by performing permutation testing [33] which involved randomising the class assignation 300 times, and for each random assignation, the two-step bootstrapping process was performed. The statistical z-test [34] was employed to determine the significance (*p* < 0.05).

## Results and Discussion

### Univariate Analysis

**Fig 1:**
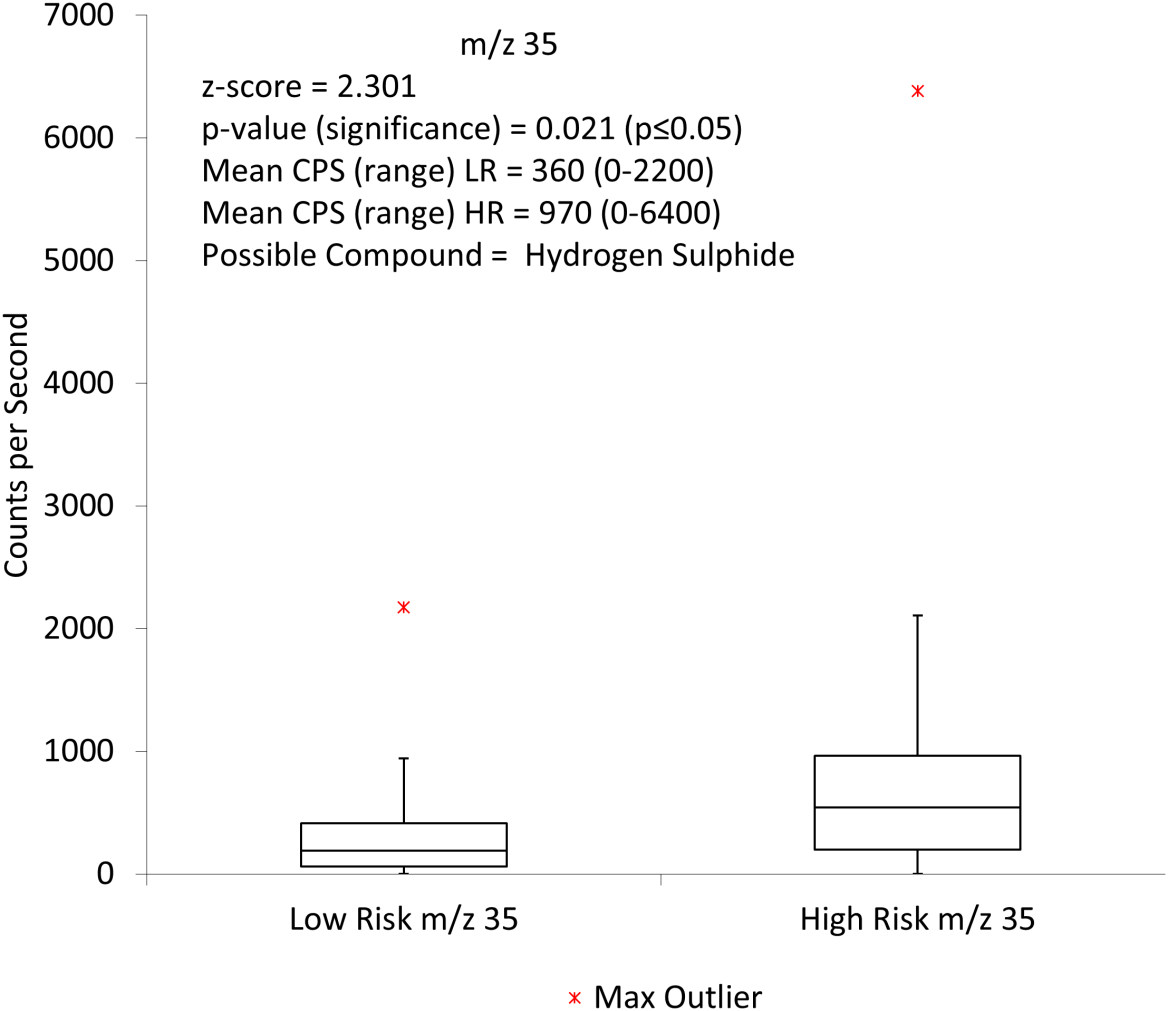
Box and whisker plot showing m/z 35 when comparing high risk to low risk faecal headspace samples for colorectal cancer with the H_3_O^+^ precursor ion. CPS = counts per second, LR = low risk, HR = high risk.

**Fig 2:**
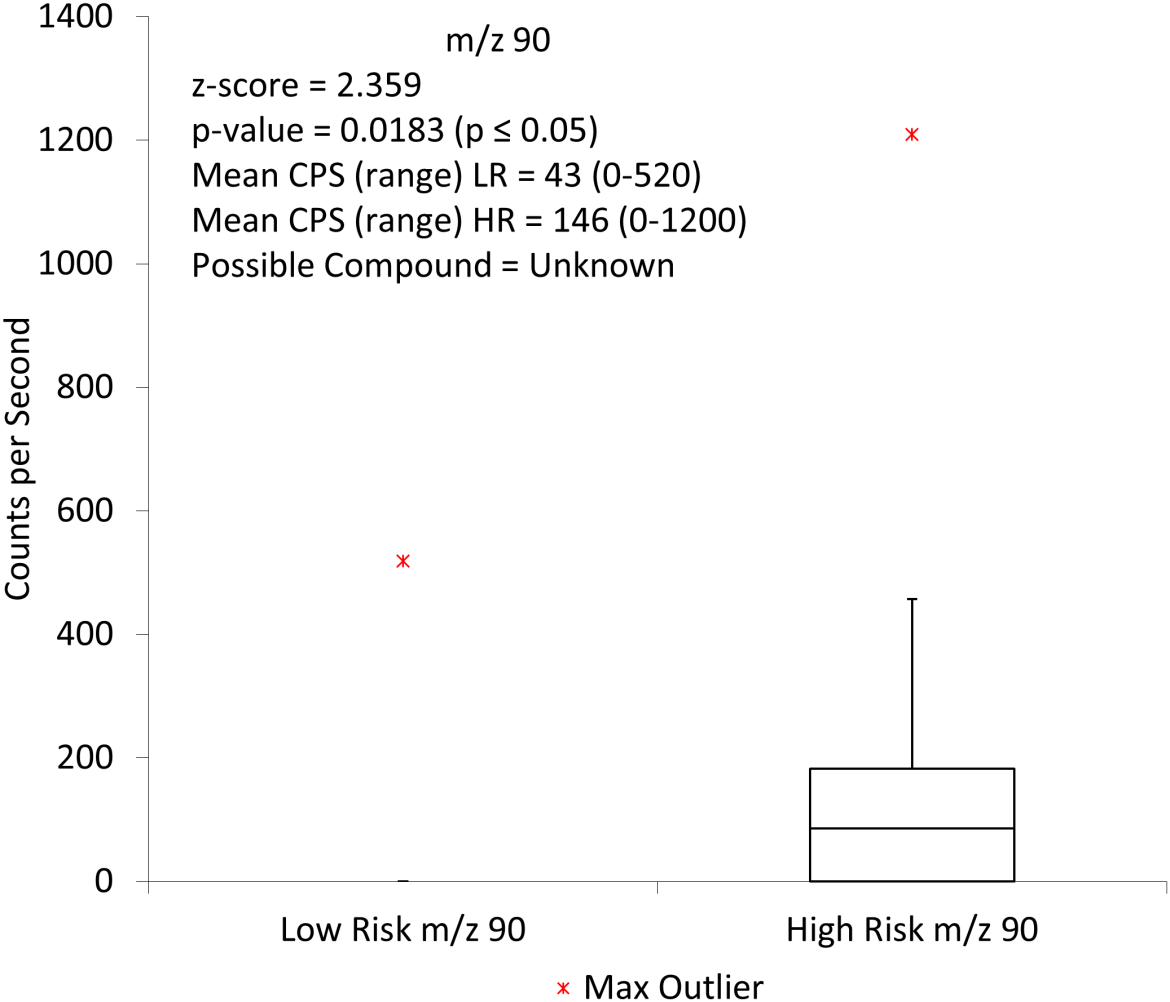
Box and whisker plot showing the distribution of low risk and high risk samples for m/z 90 using the H_3_O^+^ precursor ion. CPS = counts per second, LR = low risk, HR = high risk.

Initial comparisons using the Mann-Whitney U test analysis and the H_3_O^+^ precursor ion showed two ions to be statistically significant. These were m/z 35 (Fig 1) and m/z 90 (Fig 2)

**Fig 3:**
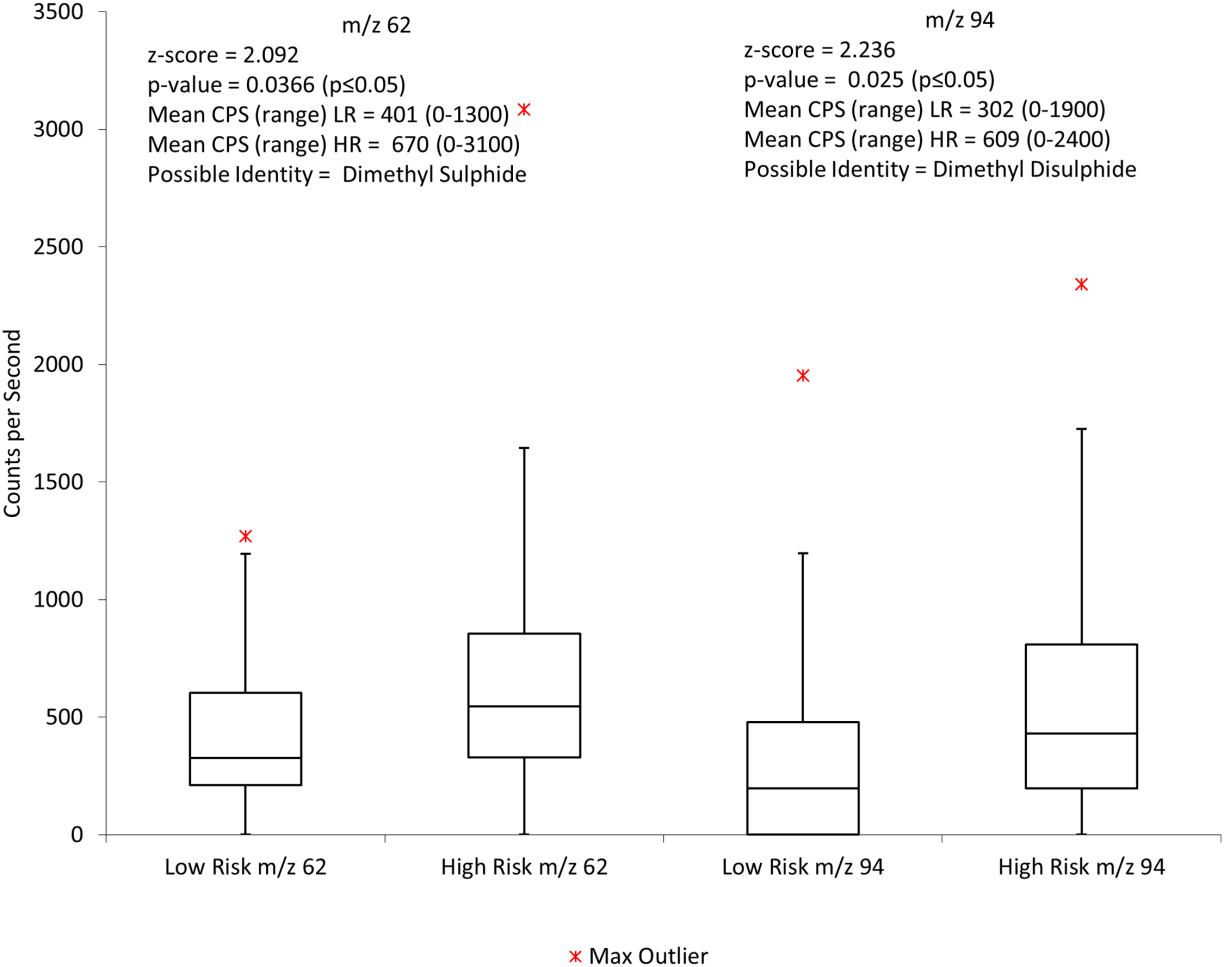
Box and whisker plot showing the distribution of low risk and high risk samples for m/z 62 and m/z 94 using the O_2_^+^ precursor ion. CPS = counts per second, LR = low risk, HR = high risk.

When using the NO^+^ precursor ion, no ions were found to be statistically significant. Finally the O_2_^+^ data were analysed using Mann-Whitney techniques and two ions were found to be statistically significant. These were m/z 62 and m/z 94. Fig 3 illustrates the significant m/z values found and their corresponding identities and values.

One of the tentatively identified VOCs is hydrogen sulphide. As a low molecular weight compound, it is easier to identify with SIFT-MS due to fewer potential other overlapping ions, and there is a reasonable likelihood that m/z35 is indeed H_2_S. Hydrogen sulphide has been widely discussed in regards to the gut and colorectal cancer. It is now well known that hydrogen sulphide is endogenous and is produced by both enzymatic reaction and microbiota in the gut [35]. The concentration in human faeces is seen to be at ‘normal’ levels when it is within the range of 1.0–2.4 mmol/kg [36]. At lower concentrations hydrogen sulphide produces a positive biological effect but at higher levels, it becomes toxic. Interestingly it does not require a specific receptor for intracellular signalling and binds to the haem molecule. The important effect of hydrogen sulphide is its binding to cytochrome c oxidase [35, 37, 38] which is the crucial mechanism responsible for its toxicity. Binding of oxygen to cytochrome c oxidase is inhibited by hydrogen sulphide non-competitively. This inhibition causes a reduction in cellular adenosine triphosphate (ATP) which subsequently has a direct effect on the ATP sensitive potassium channels. The activation of these channels plays a major role in the balance of the biological effects of hydrogen sulphide. Persistent colonisation by sulphate reducing bacteria and presence of hydrogen sulphide in the gut and faeces have been shown to be present in patients with ulcerative colitis as well as colorectal cancer [39, 40, 41] and have been implicated in the role of generating DNA damage at the genomic level [34]. Failure of colonocytes to differentiate appropriately may mean that they are more exposed to hydrogen sulphide in the lumen. The resulting effect of this may be inflammation in the gut and cell death as seen in ulcerative colitis. Continual irritation in this manner may well then lead to the genetic changes that ultimately cause colorectal cancer [42]. The mechanism by which this damage is thought to occur is via the disruption of the balance of apoptosis, proliferation and differentiation in the intestinal epithelium. These data indicate that hydrogen sulphide could be an important factor in the progress, development and diagnosis of colorectal cancer.

### Multivariate Analysis

On analysis with PLS-DA, the best resulting data were achieved by combining data sets using H_3_O^+^, NO^+^ and O_2_^+^ using PLS-DA following feature selection via the student *t* test (STT). Here an overall classification accuracy of 75% was achieved which had a specificity of 78% and a sensitivity of 72%.

**Fig 4:**
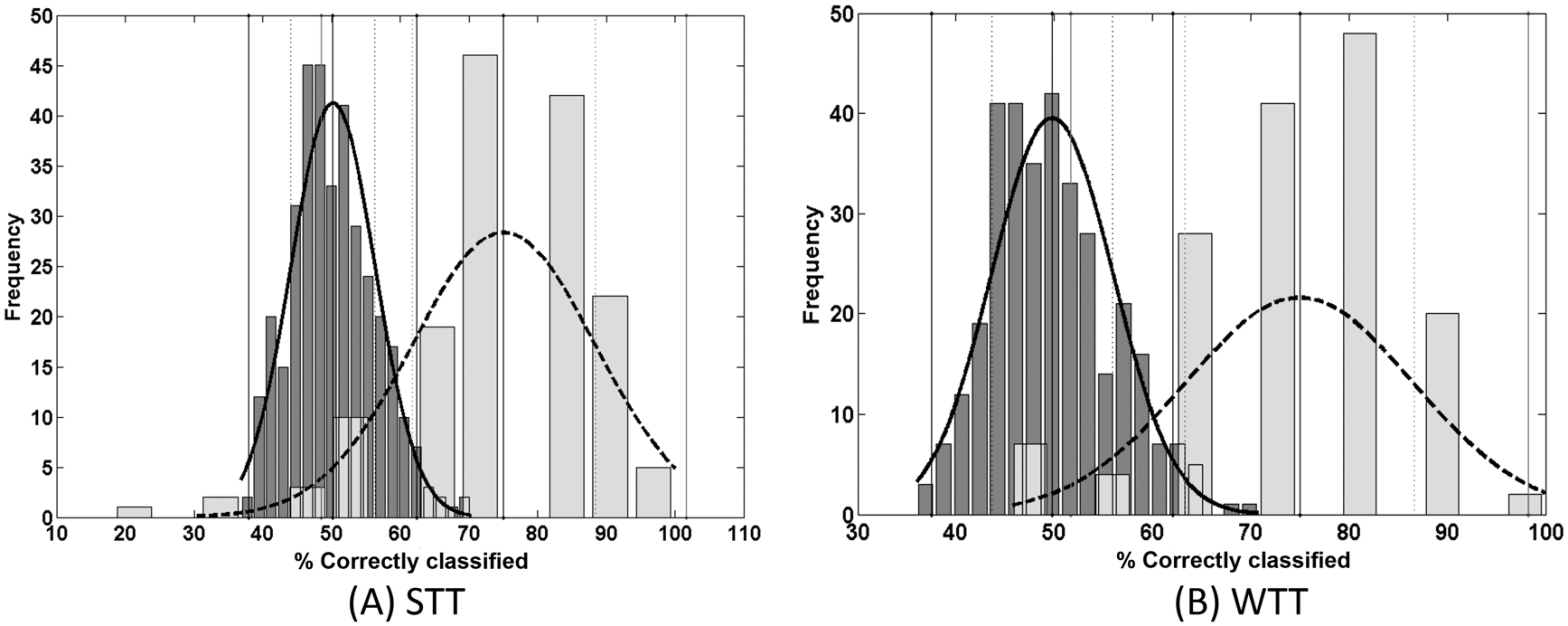
Permutation test distribution (dark grey (n = 300)) and the distribution of the bootstrapping classification accuracies (light grey (n = 150)) via PLS-DA following feature selection via STT (A) and WTT (B) for low versus high risk for all precursor ion datasets combined.

PLS-DA following feature selection with the Wilcoxon T test (WTT) also produced a promising result with a classification accuracy of 75%, a specificity of 79%, and a sensitivity of 70%. The respective distributions are shown in Fig 4.

The two-tailed z-tests produced calculated p-values of < 1.0 × 10^−6^ for STT (Fig 4A) and WTT (Fig 4B) at α = 0.05. As the calculated p-values are much less than α, the Null Hypothesis is rejected which therefore proves that there is a significant difference between the two groups, i.e. low and high risk.

**Fig 5:**
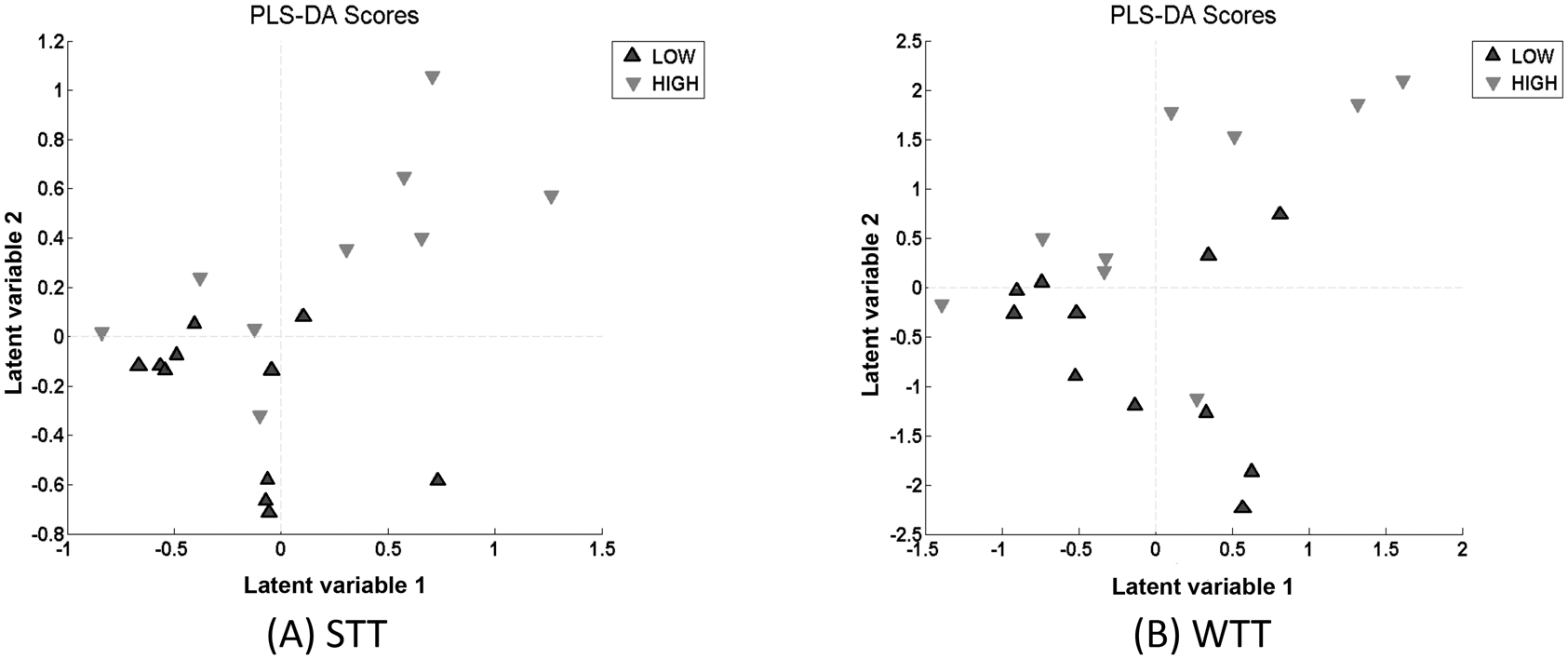
PLS-DA scores which show distinction between low and high risk extracted from the optimal models following feature selection via STT (A) and WTT (B).

Further analysis of the optimal PLS-DA model resulted in the generation of PLS-DA scores and loadings. The scores demonstrate how the high and low risk groups have been distributed and are shown in Fig 5 for STT and WTT. The loadings permit the individual m/z values to be identified that contribute to the distinction between the low and high risk samples. These values are shown in Table 2.

**Table 2:** m/z values suggested by the PLS-DA loadings when combining the precursor ions together for analysis using PLS-DA following feature selection with STT and WTT.

STT		WTT	
M/z Value and precursor ion	Possible Compound(s)	M/z Value and precursor ion	Possible Compound(s)
m/z 18—H_3_O^+^, m/z 54—H_3_O^+^	Ammonia	m/z 18—H_3_O^+^	Ammonia
m/z 35—H_3_O^+^	Hydrogen Sulphide	m/z 20—H_3_O^+^	unknown
m/z 53—H_3_O^+^ m/z 62—H_3_O^+^	Unknown	m/z 35—H_3_O^+^	Hydrogen Sulphide
m/z 68—H_3_O^+^	Pyrrole	m/z 53—H_3_O^+^	Unknown
m/z 80—H_3_O^+^	Pyridine	m/z 81—H_3_O^+^	Acetaldehyde
m/z 90—H_3_O^+^ m/z 108—H_3_O^+^	Unknown	m/z 90—H_3_O^+^	Unknown
m/z 115—H_3_O^+^	Heptanal	m/z 111—H_3_O^+^	Octanal; propanoic acid
m/z 44—NO^+^	Isopropylamine, Dimethylamine, Methylethylamine	m/z 115—H_3_O^+^	Heptanal
m/z 103—NO^+^	Unknown	m/z 44—NO^+^	Isopropylamine, Dimethylamine, Methylethylamine
m/z 52—O_2_^+^ m/z 60—O_2_^+^ m/z 70—O_2_^+^ m/z 109—O_2_^+^ m/z 123—O_2_^+^	Unknown	m/z 104—NO^+^	Propanoic acid, methyl acetate, ethyl formate
		m/z 50—O_2_^+^ 52—O_2_^+^ m/z 60—O_2_^+^ m/z 61—O_2_^+^ m/z 70—O_2_^+^ m/z 109—O_2_^+^	unknown

Fig 5 shows that the distinction between the low and high risk is captured in the Latent variable 2 (LV2) axis for both STT and WTT which is supported by the fact that the optimum number of latent variables (LVs) which produced the optimum models in both instances was 2.

### Interpreting the results

Following the use of multivariate data analysis on the SIFT-MS data, the analytical method and the technique applied to it showed a 75% correct classification to put samples into either their high risk or low risk group, when all precursor ions were combined in the analysis. This along with a specificity of 78% and a sensitivity of 72% means that overall this method is better at screening for colorectal cancer than the FOBT. The data proved to be particularly of interest, due to the fact the colorectal samples were taken after an initial positive FOBT, and after colonoscopy had been performed. As the FOBT had already been performed and classed all the samples as ‘positive’, then by carrying out the analysis following this, any ability to separate the samples in the correct groups demonstrates an improvement over the FOBT screening method.

Furthermore, both univariate and multivariate data analysis of the SIFT-MS data enabled specific compounds to be tentatively identified that were significantly different between the low risk and high risk groups. One ion was m/z 35 using H_3_O^+^ which is likely to be hydrogen sulfide. As discussed earlier, it has been well established that hydrogen sulfide plays a major role in the colon and may be implicated in colorectal cancer. This may indicate a change in gut flora, and it is possible that sulfate reducing bacteria are processing increased available hydrogen, or in fact that the sulfate reducing bacteria are present in a greater amount in the bowel of the high risk group. Shen et al. [43] investigated adherent bacteria in the gut mucosa of adenoma and non-adenoma subjects. They found a significant higher abundance of Proteobacteria and significantly lower abundance of Bacteriodetes in the adenoma patients than the non-adenoma. Sulfate reducing bacteria are found in the 5 distinct genera of the Delta subdivision of Proteobacteria phylum. Three of these genera consume partly reduced fermentation products (e.g. lactate) and reduce sulfate to sulfide, whilst the other two are hydrogen consuming [37, 44]. Red meat is also thought to increase the levels of sulfate producing bacteria which are able to use the sulfur residues from meat [45]. Higher levels of fat, red meat, and obesity have all been linked to an increased risk of colorectal cancer so could all be very relevant.

For m/z 35, the counts per second for the high risk group was 965 which had a much broader range than the low risk group, along with the higher count rate, suggesting that the m/z 35 was far more prevalent in the high risk samples. This m/z value was also identified when using multivariate analysis as a significant m/z value which further strengthens the likelihood that it may be clinically relevant. In addition, when the O_2_^+^ data were assessed using univariate analysis, two ions, m/z 62 and m/z 94 were identified which could well prove to support the theory that increased production of sulphides is more prevalent in the high risk group as they were identified as the compounds dimethyl sulphide and dimethyl disulphide respectively.

The other two compounds that were responsible for separating the groups using multivariate statistics were ammonia and acetaldehyde. M/z 18 and 54 were significant ions; m/z 18 being NH_4_^+^, and m/z 54 being NH_4_^+^.2H_2_O. These ions were higher in the high risk group than the low risk group. As previously mentioned, ammonia is the product when undigested protein that reaches the colon is fermented by the microflora. High levels of ammonia have been reported to have a range of toxic effects which include enhancing cell proliferation and favouring the growth of malignant cells [46]. When all three precursors were analysed combined, none of the acetaldehyde m/z values were found to be significant with PLS-DA and STT but m/z 81 (which is protonated acetaldehyde with 2 water molecules) was significant using PLS-DA and WTT. The potential importance of this compound in colorectal cancer is that it is a breakdown product of alcohol (ethanol). Alcohol is shown to be a risk factor for colorectal cancer [47], and if increased amounts of alcohol are being produced, or if bacteria in the gut are producing increased volumes of alcohol—subsequently leading to increased acetaldehyde—then it is likely this will cause damage. Acetaldehyde is broken down by alcohol dehydrogenase and is usually quickly metabolised by an aldehyde dehydrogenase to acetate. However in the gut, it can locally accumulate when the local microbiota oxidise ethanol and there, if folate is present, the acetaldehyde degrades it and can cause folate deficiency [48]. These results would suggest that either alcohol consumption should be reduced, or again that the balance of microbiota needs to be assessed to rebalance the breakdown of products that may lead to adverse effects.

Other compounds tentatively identified include pyridine and pyrrole. When looking at pyridine alone, no reported link is found with colorectal cancer, however the heterocyclic amine (HCA) 2-amino-1-methyl-6-phenylimidazo[4,5-b]pyridine (PhIP) has been widely identified as implicated in the aetiology of human colorectal cancer. Heterocyclic amines are formed during the cooking of protein rich foods such as meat, especially by high temperature methods (above 150°C) such as grilling, barbequing and pan frying. PhIP is seen to be of greater significance in regards to colorectal cancer because it is the predominant HCA found in cooked meats [49,50]. Heating meat creates various imidazoquinoline, imidazoquinoxaline and imidazopyridine compounds which are potent and highly mutagenic towards some strains of Salmonella typhimurium [51]. Conversely some pyrrole pigments have been identified that have carcinogen adsorption properties, which include hemin and chlorophyllin. In some studies antimutagenesis has been found with these and other pyrrole pigments both in vitro and in vivo [52].

One note of caution in dealing with the analysis of VOCs from fecal samples is that because patients produce fecal samples in their own time, and not always at clinic (as would be the case with urine or blood samples, for example), there is some uncertainty as to how samples are handled and stored prior to their return to the clinic/lab and then appropriate storage. Patients may be given advice on the appropriate way of handling and storing samples, but researchers have no way of knowing whether this has been done. This may cause variability in the quality of samples and thus reduce the effectiveness of this otherwise promising technique.

## Conclusions

Overall, what all the data suggest is that a difference can be predicted between the high risk group and the low risk group just by employing the multivariate model between the known high risk and low risk samples. This model could then be employed on unknown samples to predict if they would likely fall into the high risk or low risk group, based on their overall ‘metabolic’ SIFT-MS profile. This could then be useful in predicting whether someone is going to have or get colorectal cancer, and this method is likely to produce far fewer false positives than FOBT, thus reducing the number of unnecessary colonoscopies.

What could also then be added to strengthen the overall clinical picture is that the sample could then be analysed to specifically look for the compounds discussed and if these presented above a certain concentration, they would all suggest further investigation may be more valid for colorectal cancer. In particular, hydrogen sulphide may be a significant marker to aid in the screening or diagnosis of colorectal cancer.

## Supporting Information

S1 Table A table of the SIFT-MS data used to generate the findings reported here is available in Microsoft Excel format. (XLSX)
